# Asthma research and practice: a new journey begins

**DOI:** 10.1186/s40733-015-0005-3

**Published:** 2015-06-04

**Authors:** Juan C. Celedon, Giovanni Passalacqua, Giorgio Walter Canonica

**Affiliations:** 1 Division of Pediatric Pulmonary Medicine, Allergy and Immunology, University of Pittsburgh, Pittsburgh, Pennsylvania USA; 2 Allergy and Respiratory Diseases, Department of Internal Medicine, IRCCS San Martino Hospital-IST-University of Genoa, Genova, Italy

As co-Editors in Chief (and Professor Canonica as President of Interasma), it is our pleasure to welcome our colleagues to this first issue of the journal *Asthma Research and Practice (ARP*) [1]. This will be an open-access online journal, and thus easily accessible to a large audience. The aims and scope of *ARP* will be to publish original research articles and state of the art reviews focusing on risk factors, diagnosis and management of asthma at all ages. Since asthma is a disease that comprises diverse endotypes and is often accompanied by co-morbidities, we expect and welcome manuscripts on topics such as “obese asthma”, exercise-induced asthma, asthma-COPD overlap syndrome, occupational asthma, and others [2, 3]. Although existing journals in fields as diverse as paediatrics, internal medicine, allergy and pulmonology include articles on asthma, we believe that a journal entirely dedicated to asthma is both valuable and necessary. Moreover, we are committed to have a section devoted entirely to pediatric asthma, one of the most common chronic diseases of childhood in industrialized nations.

The title of this new journal include the word “practice” to emphasize our explicit intent to address practical aspects of asthma diagnosis and management, which are of greatest interest to the physicians and health care professionals caring for millions of children and adults with asthma worldwide. Thus, the Editors and the Editorial Board of *ARP* are committed to link mechanistic and practical knowledge of asthma in future years. We want to emphasize the strict cooperation between the Journal and Interasma [4]. This latter organization, founded in 1954 and currently chaired by Prof. G.W. Canonica, has the main missions of bridging the gaps between academy and clinical practice and of establishing an interdisciplinary forum on asthma worldwide. Notably, this mission is largely in agreement with that of *ARP*.

All manuscripts submitted to *ARP* will undergo a thorough peer-review process by physicians and scientists with relevant expertise. The Editorial Board [5] of *ARP* is committed to ensure a fair but reasonably expeditious peer-review process, followed by immediate online publication of accepted manuscripts as free articles, which will be accessible in PubMed. The Editors and the Editorial Board further commit and expect that *ARP* will rapidly get an Impact Factor that reflects the quality and value of this journal.

We would like to express our profound gratitude to our dear colleague and friend, Professor Carlos Baena-Cagnani. Carlos conceived and enthusiastically supported the preparation and launching of this journal, which is and will always be a tribute to his memory.

The first publications in *ARP* include two original research articles and a comprehensive review. The first article reports the findings of a Phase I clinical trial of vitamin D supplementation in patients with cystic fibrosis and allergic bronchopulmonary aspergillosis (ABPA), a condition that causes significant morbidity in patients with asthma [6]. Although preliminary by design, this Phase I trial demonstrates the efficacy of the tested dose of vitamin D (4,000 IU/day) in both achieving vitamin D sufficiency and reducing Th2 immune responses relevant to ABPA and other allergic conditions such as asthma. The second article reports the findings of an Italian questionnaire-based survey about respiratory allergy, as described by patients referred to pharmacists for their symptoms [7]. This aspect is of special interest, since pharmacists are usually the first-line referral. The Authors found that 11 % of patients who accessed pharmacies had only lower respiratory symptoms, but that a large fraction of the population had co-existing upper and lower respiratory symptoms. Notably, about 50 % of patients had not received a medical diagnosis, and thus self-management of repiratory symptoms was common. In spite of the limitations of the questionnaire-based design, those findings highlight the problems of under-diagnosis and under-treatment. The third article reviews current literature on the increasingly common “obese asthma” phenotype, including potential causes, modifiers and common co-morbidities; impact of overweight or obesity on asthma control; growing evidence of distinct endotypes of “obese asthma”; and treatment of patients with co-existing obesity and asthma [8].

We conclude by inviting all of our esteemed colleagues to submit their work to *Asthma Research and Practice*, while also welcoming their suggestions to improve the quality and reach of the journal.
